# Consumer Experience and Omnichannel Behavior in Various Sales Atmospheres

**DOI:** 10.3389/fpsyg.2020.01972

**Published:** 2020-08-07

**Authors:** María Dolores Reina Paz, Fernando Jiménez Delgado

**Affiliations:** Department of Business Economics and Accounting, National University of Distance Education (UNED), Madrid, Spain

**Keywords:** experience, purchasing process, digital atmosphere, omnichannel, consumer behavior

## Abstract

The conceptual framework for our analysis is the approach to environmental psychology first introduced by different authors in 1974. Subsequently in 1982 this concept was applied to retail outlets so as to better understand the effect on consumers of atmospheric stimuli experienced in a physical store. Following the developing trend from traditional marketing to new online sales channels, various authors have sought to transfer and validate these theories with virtual outlets or e-stores, so as to validate them in a context of e-commerce. Our study thoroughly analyses and reviews the most widely accepted models in the study of the influence of sales atmospherics on consumer behavior in physical store environments, and the adaptation and application of such models to today’s omnichannel shopper behavior, where shopping environments combine physical sales settings with new digital sales atmospheres.

## Introduction

The internet offers companies a great opportunity to enlarge their customer base by marketing their products and services online, in what is known as “e-commerce.”

In this regard many companies which had focussed their efforts on traditional physical channels are now also adding online channels in a multichannel strategy. But what is interesting is that such companies do not abandon their physical channel but rather adopt an “omnichannel” strategy as a strength vis-à-vis purely online firms (“pure-players”), which paradoxically are also starting to set up physical environments or “experience” stores (Blazquez et al., 2019).

Being present in multiple channels offers greater exposure and market reach, but if the customer is the same, companies must start to consider the congruence of customer experience when switching from one channel to another and whether they are maintaining branding uniformity and consistency across specific marketing attributes in each channel so as to enhance value in the consumer’s shopping process (Juaneda-Ayensa et al., 2016).

The growing importance acquired by the environment in which the consumer’s patronage occurs means that this analysis is a chance to explore the evolution and adaptation of the various shopping behavior models based on the stimuli received by consumers from the setting in which their general shopping experience takes place. This is especially so given that we have known for some time that “an arousing store environment or atmosphere combined with a pleasant shopping experience have a positive effect on consumers’ willingness to buy” (Bitner, 1992).

Some authors describe a type of hedonic buying referred to as “adventure shopping” (Arnold and Reynolds, 2003) in which the need the consumer seeks to satisfy is prompted precisely by these cues that shoppers receive from their environment during the purchase process – cues that may involve one or more sensory organs and which make the mere act of “exploring” or walking about a store a pleasant experience, well beyond the merely utilitarian outcome to be procured by the purchase of a particular product or service.

It is for this reason that considering the shopping reality in a virtual environment is significant, as some of these senses are to some extent restricted and this sensory deficit often needs to be offset with extra input and enhanced features so as to give the consumer a pleasant experience with hedonic purchases also in this channel (Jang et al., 2018).

Technological advances concerning ease of use, new features and increasingly sophisticated security and reliability in the online environment have made new tech more acceptable to users. All this has driven research and development with a view to further technological progress for improving the shopping experience, which is undoubtedly a driver of e-commerce as an alternative or complement to traditional retail channels. In this regard, new graphic environments, improved hardware and software performance and greater internet bandwidth allow hyperrealist environments (in 3D) to be created such as till now were reserved for physical stores (Hassouneh and Brengman, 2015).

Regarding these hyperrealist environments, we find that in recent years there has been great growth in virtual reality scenarios with various designs and goals but all based on the idea of giving consumers a more realistic and immersive experience^1^, with acute awareness of the reality in which the user is interacting.

Initially many of these experiments emerged from the world of videogames, when titles such as “Minecraft” were hugely popular with the gaming community, often creating a subculture among gamers.

Also initiatives aimed at the business world such as “Second Life” sought to create a virtual economy parallel to the real-world one, connecting the two worlds via a value creation model using a “virtual other me” better known as an “avatar” (Arakji and Lang, 2008).

In recent times various business initiatives have sought to combine both “worlds” – that of traditional physical trade and the virtual one, with virtual reality experiences at the point of sale (POS). Especially notable are those developed in the sphere of apparel, the fastest-growing product category on the internet in recent years (Blázquez, 2014).

## Digital Atmospheres

Since the 70s, academic research has paid increasing attention to sales atmospheres. Kotler was one of the first authors to describe the use of “atmospherics” as an attempt to design buying environments liable to produce emotional effects in the buyer to enhance his/her likelihood of purchase. Music, colors, smells, lighting, etc., are atmospheric cues, and given the internet’s multimedia character, ever more websites are using such cues. Now how can we predict their effects in the new environment of e-commerce? An increasing number of studies have been analyzing elements of website design. For it seems clear that a website, as an environment, can influence responses and behaviors with its design.

Building on Kotler (1973) concept of store atmospherics, Childers et al. (2001) have coined the term “webmosphere.” Dailey (2004) defined the concept as “the conscious designing of web environments to create positive effects (e.g., positive impressions, positive cognitions) in users in order to increase favorable consumer responses (e.g., views, browsing, etc.).” According to Childers et al. (2001) the webmosphere includes structural design attributes (frames, links, pop-up windows, etc.), media dimensions (graphics, text, audio, video, etc.), and site design (organization and grouping of products). For this line of research, the design of e-stores or virtual stores plays the same role as that of brick-and-mortar stores (Liang and Lai, 2002) and so it seems reasonable to expect the design of e-stores (e.g., homepage look and design) to be liable to significantly influence consumers’ online perceptions, and consequently their willingness to buy in such stores (Baker et al., 2002).

Yet the webmosphere’s actual influence on consumers remains a subject of debate. For some researchers, screens are the key to establishing the webmosphere’s dominance of atmospherics, along with the capacity to represent text, graphics (images, animations, videos) and sounds satisfactorily, so as to create genuine “online atmospheres” (Galan and Gonzalez, 2001).

Thus the three PAD variables (pleasure, arousal, and dominance) initially introduced by Mehrabian and Russell (1974) may equally describe “virtual or physical behavior” (Koufaris et al., 2001). For example, enjoyment of online shopping increases where consumers participate actively, interacting with the online store environment.

But some researchers take a more cautious view. They note that various atmospheric cues, such as temperature, smell and touch, are not yet available on the internet (Eroglu et al., 2001) and wonder whether the use of tools such as video may contribute to enjoyment or to frustration, in view of download times (Childers et al., 2001). But they acknowledge that e-commerce has its own features (or even advantages) relative to traditional commerce, such as flexibility in time and space, or downsizing the sales environment to the area of a screen.

Where there seems to be agreement in the academic community is that in the design and environment of e-stores there are highly significant factors beyond merely practical ones, to do with enhancing the experience and appeal in the presentation of products on e-commerce sites (Detlor et al., 2003).

Also how crowded or “atmospheric” a store is in view of the presence of other consumers can influence patronage intentions (Baker et al., 2002). Thus a pleasant or attractive store atmosphere prompts consumers to spend more time in the store, resulting in a greater likelihood of buying goods there (Donovan et al., 1994).

In short, there is a broad consensus as to the key role played by the store environment or atmosphere and its influence on the shopping process.

As to stimuli at the POS or in-store merchandising, today these go far beyond conventional signage or POS artifacts. For instance, stimuli such as the music selected to be heard in the store (the style or genre, the volume it is played at, its beat, etc.) have been shown to influence both consumer mood and behavior (Santos and Freire, 2013).

Other atmospheric cues such as choice of color scheme (Bellizzi et al., 1983), lighting (Areni and Kim, 1994), tidiness (Pinto and Leonidas, 1995), and the store’s scent or fragrance (Hirsch, 1995; Mitchell et al., 1995; Smith and Burns, 1996; Peck and Childers, 2003) are likewise all “merchandising” stimuli affecting consumers’ mood, attitude and behavior.

And as new merchandising items are introduced in physical or traditional sales channels, so are they in digital channels, where moreover the digital environment itself offers new formulas facilitated by technology, such as interaction enabled by social networks as a form of merchandising, as we interact with the products offered on a site.

In this regard, merchandising in a virtual sales environment plays a large role in shoppers’ decisions. For example, messages such as “other users bought this item” based on buyer decisions. Or the number of references or the assortment and arrangement at the top or bottom of the page, requiring the consumer to scroll or click through the online store (Lorenzo et al., 2008), or the use of videos presenting the product – all are cues influencing the buying process (Gurrea and Sanclemente, 2014).

## User Experience

Currently there is growing interest in everything to do with consumers’ experience with brands (Pine and Gilmore, 1998) and how the new channels available to consumers are evolving as alternatives for shopping (Shankar, 2014).

In Pine and Gilmore (1998) devised a classification of realms of experience based on the nature of experience factors and how they function and interact.

In their model there are two key dimensions: customer participation (active or passive) and relationship with the environment (immersive or absorbent).

1. Active participation refers to situations in which consumers directly influence performance, such as on playing a computer game or participating in a team sport.
2. Passive participation refers to the converse situation in which customers do not affect performance, such as on watching TV or seeing a theater play.
3. In the environmental (ambient) dimension, immersion refers to being part of the experience (such as in first-person online gaming on a PC or console).
4. Absorption refers to situations in which the consumer’s attention is engaged in bringing an experience to mind, such as on seeing a film in the cinema.

Three-dimensional (3D) experiences have been adopted by e-commerce websites both to entice consumers to visit the site and to encourage online shopping, and also to enhance satisfaction, while building loyalty and turning consumers into regular visitors or customers (Fiore et al., 2005).

Regarding 3D virtual stores, some authors (Barnes and Mattsson, 2008) have shown in their studies that viewing products on screen in 3D helps to create brand awareness, and also allows users to experience facets of the virtual product not accessible in a 2D environment (Guidi et al., 2010).

In short, interface design that creates a vivid experience (i.e., akin to sensory experience and behaviors experienced with an actual product) positively affects approach responses to the product. These approach responses to a product or site are the result of utilitarian value (time saving, control, better product information) and hedonic value (enjoyment) generated by interactivity. The combination of the two values (utilitarian + hedonic) greatly conditions consumer responses, as shown by the authors Koufaris et al. (2001).

There are many studies on the new virtual shopping channels now available to consumers, and in particular on the validity and effectiveness of cues for stimulating purchases in a physical outlet when transferred to an online channel (Ha et al., 2007; Breugelmans and Campo, 2011; Ha and Lennon, 2011).

In brick-and-mortar outlets, authors such as Zorrilla (2002) have confirmed that “variables such as music, scent or lighting affect consumers to such an extent that they may then chose to extend their visit and count the store among their favorites.”

And in the same author’s words, “we believe these are variables that retailers should keep in mind when planning, just as when selecting a product range or pricing policy. Indeed they should develop a consistent offering of commercial variables in which the staging of products at the POS highlights their value, communicates the store’s personality and gives added value to the shopper’s visit. Experience will play a key role in the perception and image of the store and its relationship and bonding with customers” (Zorrilla, 2002).

Also it is paradoxical that whereas shopping experience in a brick-and-mortar environment always occurs in a 3D arena, almost all online retailers (“e-tailers”) opt for browsing with 2D imaging in their web stores, yielding a highly different brand image and experience (Kwon and Lennon, 2009).

Combining the above two concepts, i.e., brand image and experience, virtual consumer experience has been defined in marketing literature as “psychological and emotional states that consumers undergo on interacting with products and brands in a 3D environment” (Li et al., 2001).

Thus, speaking of consumer experience in a virtual environment, we may conclude that “virtual experiences” are computer-generated scenarios simulating physical ones, liable to generate a convincing experience. We also know that increased tangibility in “atmospheric cues” on the internet results in positive evaluation by consumers and reduces the risk perceived by potential online buyers, as found by Koernig (2003).

This tangibility effect taken to the limit, i.e., giving the sensation that the consumer is really present in the virtual environment, is known as “telepresence.”

Telepresence is achieved as a function of “vividness” (technology’s ability to produce a rich sensory environment) and “interactivity” (our degree of control over the manipulation of form and content) (Steuer, 1992; Hoffman and Novak, 1996). So we may infer that the more realistic virtual product experiences and brands are, the better they will be perceived, and so the more users will experience telepresence (Li et al., 2001; Chin and Swatman, 2005).

Regarding the role played by the vivacity of information in a virtual experience, there is a large literature on the influence of site design variables in the subject’s shopping experience (Lorenzo et al., 2008). Most studies have focussed on contrasting static 2D elements (images) with dynamic 2D elements (videos), or static 2D designs (images) and 3D designs static (images) (Gurrea and Sanclemente, 2014). Other authors (such as Baek et al., 2015) have proposed models contrasting a static 2D scenario with an immersive 3D scenario, giving a highly realistic feeling and allowing study subjects free interaction with their environment, promoting the sensation of “being there,” i.e., of telepresence (Hassouneh and Brengman, 2015).

Further to what was said above on the role of images in generating telepresence, research with various imaging techniques also underlies new disciplines such as “neuromarketing,” whose origin is attributed to Paul Lauterbur and Peter Mansfield in the early 1990s, though it was the German professor Ale Smidts (at Erasmus University) who coined the term “neuromarketing” in 2002 (Krajnović et al., 2012).

Authors such as Darren Bridger, who has been working in the field of neuromarketing for more than 16 years and has mounted countless market research projects in various sectors, assert that the advantages of research with neuromarketing techniques go beyond those available with conventional models (Bridger and Noble, 2015).

Specifically Bridger argues that neuromarketing has two key advantages: (1) It provides new perspectives in user experience: advertising media today are influenced by new ideas and new perspectives. Neuromarketing offers novel metrics because it takes a perspective very different from that of traditional research models. To cite just one example, more subliminal effects can be gauged in terms of consumers’ attention, emotions and memory responses to creative designs (such as printed adverts) and videos (such as TV and web adverts). Among the various creative projects often proposed in advertising, neuromarketing research focuses on the more experiential aspects of the buying process. Its methods often help to pinpoint the triggers behind these emotional reactions.

(2) Neuromarketing measures the effects of arousal in the consumer’s mind when faced with various imaging techniques. When an item such as a poster, product or e-commerce site is viewed, associations of ideas arise and interact in the mind. Some of these ideas, which can be measured by neurological methods, are linked to the notions and emotions aroused in individuals by advertising or brand logos. Some often involve mental cues with no conscious awareness. For example, behaviors in a person buying a luxury product may be triggered by certain methods of imaging or depiction. These effects aroused in the consumer’s mind in response to cues are analyzed by neuromarketing in what is called the “neurobiology of learning.” The outcome of a decision, behavior and even learning depends on the quantity and quality of neuronal connections formed from cues in information and experience, along with the ability to perceive more and better information and experiences, and brain processing capacity, which depends in turn on prior stimulations (Braidot, 2005).

## Omnichannel Buying Process

Today it is increasingly common for consumers to use various shopping channels when choosing a product. Depending on the type and category of product or service, the percentage sold in a physical store as opposed to online will vary, but what is found increasingly is the use by consumers of online channels in conjunction with brick-and-mortar stores.

In most cases, potential consumers prefer to use online channels for research in the early phases of a purchase decision (online research), even if they finally make the purchase/transaction at a physical outlet where they will preferably have access to the preselected product (offline purchase). This effect is known as Ro-Po or “webrooming” (Puccinelli et al., 2009).

This contrasts with other product categories, like consumer electronics (audio/video) or home IT (PCs/printers) or handheld devices (tablets/smartphones), for example, where consumers are more willing to take every step on the path to purchase (research, comparison, selection, and transaction) without leaving the online channel; or even in some cases, after seeing the product on show in a brick-and-mortar store, they opt to buy it in an online one. This effect is also called “showrooming” (Melis et al., 2015).

With this new reality, the study of omnichannel consumer behavior should take account not just of factors linked to the intrinsic characteristics of the product and their influence on the channel chosen by the consumer (physical or virtual), but also the intrinsic profile of the online shopper or “virtual consumer” as against the “physical store” shopper profile.

Thus Lorenzo et al. (2008) that “including and blending various online experience factors (i.e., usability, interactivity, trust, aesthetic aspects, and marketing mix) in the design of a virtual store results in the creation of multiple e-store designs, thereby triggering various perceptions in users which knock on to their shopping behavior.”

It has been shown that a consumer’s buying process generally involves three stages (Frambach et al., 2007) identified as “pre-purchase,” “purchase,” and “post-purchase.”

The extant research also shows that consumers switch or “skip” between the various online and offline channels when working through these stages (Ahuja et al., 2003). Such behavior is known as “omnichannel” or “interchannel” and is more significant where the purchasing of products or services is more complex.

It seems that in the pre-purchase stage, in which consumers mainly seek information, they have different requirements of the marketing channel relative to the purchase stage, in which consumers make the final transaction (Neslin et al., 2006; Verhoef et al., 2007).

Accordingly it is important for companies seeking to reach consumers via both online and offline channels to understand their needs and their preference by channel at each stage of the shopping process, so as to exert proactive influence and to determine when and how to act at each stage in each channel so as to improve the consumer’s overall (“omni”) experience.

Whereas in the case of planned shopping (or for durable consumer goods, where the outlay is bigger), there are normally various sub-stages within the three main stages described above, this is not so in the case of impulse buying or fast-moving consumer goods (FMCG).

Indeed some authors suggest that the pre-purchase stage (in which information is gathered, comparisons are made and a short-list drawn up) may not exist for such heavily consumed products, and some studies find that about 76% of customers take the buying decision actually at the POS when “in the aisle” (Mahoney, 2012). It is at this decisive point, when the consumer pauses in the aisle by the product shelves, that what some authors call “the moment of truth” occurs, largely conditioned by the stimuli received at this impulsive point (product arrangement, packaging, in-store merchandising, etc.).

But in products and services with more complex purchasing cycles, as shoppers work through the various sub-stages of the buying process, they are influenced by many factors, notably (1) characteristics intrinsic to consumers (e.g., demographics, psychographics) and shopping experiences (behavior patterns), (2) in-store stimuli (e.g., display cases, store shelves, merchandising, positioning, and arrangement, etc.), and (3) out-of-store stimuli (e.g., community word of mouth, comments on social networks, and apps or review site ratings), as well as learning based on observation and comparisons.

Although the stages and sub-stages of the shopping cycle apply to any product category, much of the research on “shopper marketing” focuses on FMCG, as it is with these much-used, low-cost products that more impulsive emotional considerations cued by stimuli in the sales environment determine to a greater extent what goes into the basket.

Speaking of the shopping process in the digital age, in 2011 Google came up with the ZMOT (Zero Moment Of Truth) concept as a stage or point prior to the moment of truth (MOT) mentioned above, occurring at traditional brick-and-mortar outlets.

Thus the US firm says a buyer typically starts with a “need to be met.” What is involved may be as routine as buying a carton of milk or as unusual as finding a new home, and in both cases there is a “zero moment of truth” that arises before the buyer even accesses the store where the first MOT is conventionally experienced.

This zero moment may have arisen from a search for information to meet an initial need on the internet or a Facebook post, or by word of mouth with friends or from a “story” posted on Instagram by a shopping “influencer” (Hajli et al., 2017).

As with purchase decision factors in traditional commerce, in the shopping process in the digital age we also find significant differences between planned and unplanned buying. Analyzing which sources of information are used by consumers is vital to companies, as it is these sources which marketing endeavors should target. An example of this is that shoppers increasingly use mobile devices to search for information, so the use of smartphones connected to the internet has great implications for the shopping process in the digital age, especially when such searches on mobile devices are made near or even at the POS (Mosquera et al., 2018a).

## Models of Consumer Behavior in Response to the Environment: Concluding Remarks

In the analysis of individual behavior in an environment, it was first in 1974 in that the authors Mehrabian and Russell identified three emotional states (conditioned by approach-avoidance) in response to a particular environment. These three states made up a model known as “PAD” (Pleasure, Arousal, Dominance) including the following emotional dimensions: (1) Pleasure, relating to how the consumer feels (pleased, good, happy, and satisfying feelings), (2) Arousal, relating to the degree to which the consumer is stimulated, alert or roused in the situation experienced, and (3) Dominance, relating to whether he/she feels controlled or free to act in a given situation (reactance).

The model was thus based on these three “basic emotional dimensions,” though 10 years later Russell himself mapped as many as 40 different emotional “states” under them.

Briefly the PAD model established a conditional interaction between pleasure and arousal, so in a neutral environment, moderate arousal enhanced approach behaviors, whereas with extreme degrees of arousal (very low or very high) the tendency in consumer behavior was to avoidance. In pleasant environments, the greater the degree of arousal, the more approach behavior there was. But in unpleasant environments, with high degrees of arousal, consumer behavior tended to avoidance.

Eight years later, the authors Donovan and Rossiter (1982) adapted the original PAD model, linking the study of the emotional dimensions of “pleasure” and “arousal” to that of dominance or reactance. Thus they applied the theory to the setting of a retail outlet in order to better understand the influence on consumers of stimuli from the atmosphere perceived in a physical store (Donovan and Rossiter, 1982). The work of these authors laid the basis of the model that has been applied since then in studies in the sphere of retail outlets and their atmospheric variables. This model is known as S-O-R (Stimulus-Organism-Response) and has been widely developed in the academic literature on consumer behavior.

**FIGURE 1:**
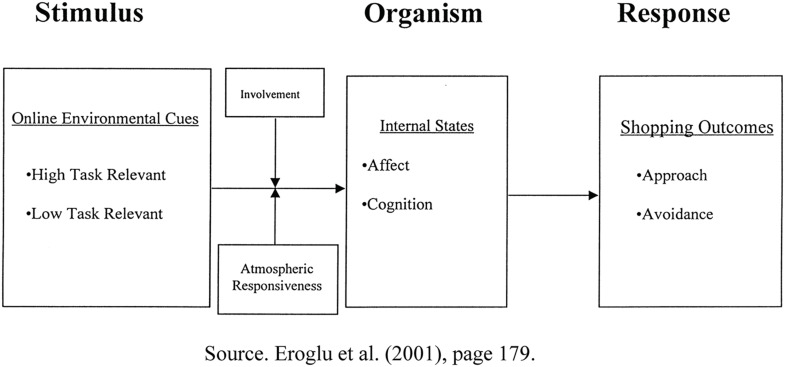
An S-O-R model of consumer response to online shopping. Source Eroglu et al. (2001), page 179.

In the S-O-R model, a stimulus is conceptualized as an influence that arouses the individual, and thereby influences internal states in the organism, as shown in Figure 1. Environmental psychology has been widely used in studies of the field of “attraction” and “repulsion” based on the S-O-R model, suggesting that environmental stimuli result in “approach” or “avoidance” behaviors.

There is an extensive literature studying the S-O-R model in a real-world setting (physical store) based on the study of various dimensions of stimuli at a store or POS (exterior, interior, design, display, and staff) and how these affect buyers and sellers and their corresponding responses or behaviors (Turley and Milliman, 2000).

Subsequently Donovan et al. (1994) again used this model to describe the relationship between environmental aspects and approach and avoidance behaviors in the relevant environment, all influenced by the person’s emotional behaviors under their various “states” stimulated by that setting.

Year later, Turley and Milliman (2000) used the S-O-R model to describe the approach and avoidance concepts as follows: “Approach behavior is a positive response to the environment, in which a person feels the need to remain in that environment and to explore it, whereas avoidance behavior is manifested when the person does not wish to remain in the store or to spend time looking around or exploring.”

Investigating the complexity of these stimuli, the study of the physical environment of traditional stores or outlets has shown that we are influenced not only by the cues we receive directly but also by other environmental factors, such as store humidity or temperature^2^, which may condition our behavior as consumers, and, for example, hasten our decision process so that we leave the store earlier (Hadi and Block, 2019).

In the context of online sales, stimuli are defined as the sum total of all the cues visible and audible to the online consumer (in the literature also called “e-consumer” or “e-shopper”).

As against the physical stimuli present in a traditional retail store, an online retail sales environment lacks some of the dimensions (temperature, smell or texture) defined by Baker et al. (2002) which stimulate the various areas linked to these senses in the cerebral cortex.

Nor do we have a visible presence of other buyers and employees (the social dimension, according to Baker) in an online retail sales environment, though their presence may be suggested by indirect indicators such as web counters, postings on site message boards or delays in access to areas of the site because the computer system is tied up with other users.

A virtual store environment (online retail sales site) evidently lacks certain features of brick-and-mortar retail settings (such as three of the five senses: smell, taste, and touch), but it has other advantages (such as flexibility in time and space) which combine to make it an ecosystem significantly different from conventional retail environments, with highly promising research opportunities for the fields of neuromarketing and neuroscience applied to business.

These differences between environments in an omnichannel scenario have required a validation of the original S-O-R model, as the reactions widely accepted by the academic community in the study of traditional atmospherics in physical premises cannot fully apply to the online sales environment.

In this regard and coinciding with the rise of e-commerce (in 2001), many studies were published to test the validity of the S-O-R model in a context of online outlets (Ha and Lennon, 2010) where stores cease to be physical spaces and the shopping experience is recreated in an “enriched” virtual setting (Eroglu et al., 2001; Ha and Lennon, 2011). The media “richness” theory (Eroglu et al., 2001) which differentiates between “lean” and “rich” media based on the quantity and quality of cues offered, can be useful in this effort. “Lean” media are characterized by unequivocal and unambiguous information while richer media contain more emotional, ornamental and emphatic features. Eroglu suggests that all screen-mediated communication is essentially “lean” given its inability to represent most of the sensory and sensual aspects present in brick-and-mortar arenas.

For the discipline of marketing in e-commerce this is quite a challenge, and the extent of leanness may be determined by the degree to which the on-screen information presented to the e-shopper is directly relevant to his/her shopping goals that may differ due to gender wise (Mosquera et al., 2018b). For instance, a shopper may go to a site looking for a pair of green trousers. They may find a picture of the trousers, a description of the fabric and workmanship, sizing information, the price and the terms of ordering and shipping. All such information would be directly relevant to the shopper achieving the goal of buying a pair of green trousers.

Alternatively, the site description may contain diverse decorative and/or vivid depictions (such as photos of people enjoying some activity while wearing the pants, striking background colors and graphics, interactive images to click on to go to the next area of the site rather than simple underlined hyperlinks), or even animations or videos (Gurrea and Sanclemente, 2014) which, while enhancing the hedonic quality of the shopping experience, may not directly provide much useful information for achieving the consumer’s shopping goals.

Concerning another aspect of the omnichannel reality, authors such as Childers et al. (2001) have extended the S-O-R model including the concept of “digital atmosphere” or “webmospherics.”

A more recent study published by Song and Kim (2012) at Oregon State University also applies this model to the visual presentation of products in the online retail trade using the variables of picture size and number of product views available.

Also for other disciplines mentioned above such as neuromarketing, the study of consumer behavior in a web environment where some of the five senses are restricted represents a challenge, as neuroscience has traditionally focussed on environments of multisensorial experience (Krishna, 2012).

Thus for example from a neuromarketing perspective, we can artificially recreate responses (of pleasure or avoidance) in a consumer who hypothetically enters a store that is more or less tidy or has a more or less varied product range, and generate a sense of telepresence “as if actually there” in a highly realistic way with new technologies, and create a truly immersive 3D atmosphere.

An alternative paradigm for approaching the study of telepresence and webmosphere is the “Technology Acceptance Model” (TAM) developed by Davis et al. (1989). This model focuses on user-machine interaction in terms of learning, usefulness and ease of use of new technologies. This same model was updated by Venkatesh and Davis (2000).

But if the aim is to not so much to focus on a framework of learning and usefulness as regards technology as to explore the variables of a sales-specific environment influencing consumer behavior, the discussion will continue to revolve around behavioral models taking account of the influence of environmental variables, such as the early models developed by Kotler (1973), Bitner (1992), Botschen et al. (1999) and other authors mentioned in our previous section, whose studies are detailed in Table 1, and the subsequent S-O-R model on which our conceptual analysis has largely been based and which continues to be revised and adjusted by many authors in keeping with the atmospheres which today’s omnichannel consumers encounter.

**TABLE 1:** Models of consumer behavior in response to the environment.

**Study**	**References**	**Findings**
An approach to environmental psychology	Mehrabian and Russell, 1974	This study laid the foundations of what would subsequently be known as the theory of consumer behavior in retail outlets. The authors are without doubt pioneers in analyzing, from the sphere of psychology, individuals’ response to influential environmental stimuli.
Store atmosphere-an environmental psychology approach	Donovan and Rossiter, 1982	Setting out from the field of psychology, Donovan and Rossiter adapt the Mehrabian-Russell model to a consumer behavior environment. This is a key study establishing the basis of the S-O-R model for all future point-of-sale research studies.
User acceptance of computer technology: A comparison of two theoretical models	Davis et al., 1989	An interesting experiment with students to explore the factors behind resistance to the adoption of new technologies, and in particular computer use. It was found that the factors of ease of use and usefulness based on a learning process result in higher ICT uptake.
An experimental approach to making retail store environmental decisions	Baker et al., 1992	These authors originally identify three environmental categories: social, design and ambient factors. Ten years later they revised their study, with more on interpersonal factors.
Store environment and consumer purchase behavior: mediating role of consumer motions	Sherman et al., 1997	The authors propose certain consumer responses and measure the relationships between environmental cues and those responses, mediated by internal states.
Welcome to the experience economy	Pine and Gilmore, 1998	Definition of the subject’s various degrees of experience with a product or service according to the levels of absorption and immersion achieved.
Atmospheric effects on shopping behavior: A review of the experimental evidence	Turley and Milliman, 2000	In this review of atmospheric variables and cues at the point of sale influencing consumers, the authors include a broad compilation of the literature on the subject with more than 60 studies classified by the variables and cues analyzed.
A theoretical extension of the technology acceptance model: Four longitudinal field studies	Venkatesh and Davis, 2000	In an extension of the original 1989 study, this second version explores user-machine interaction from the perspective of learning, usefulness and ease of use of new technologies.
Hedonic and utilitarian motivations for online retail shopping behavior	Childers et al., 2001	A key study on the importance of creating a favorable atmosphere in a web environment and of consumers being motivated when meeting their basic shopping needs and also in particular when shopping needs are hedonic.
Un cadre théorique de l’impact des éléments de conception du site web sur les réponses des consommateurs (A theoretical framework for the impact of aspects of website design on consumer responses)	Galan and Gonzalez, 2001	This study discusses the role of online atmospherics in an e-store according to the context of the visit, distinguishing between browsing for information or for leisure or for merely utilitarian purposes.
Consumer behavior in web-based commerce: an empirical study	Koufaris et al., 2001	This study links consumer experience in a web store with the ability to build consumers’ loyalty to the site and their intention to return to buy.
Atmospheric qualities of online retailing: A conceptual model and implications	Eroglu et al., 2001	This is one of the first studies which, given the lack of a specific framework for consumer behavior in an e-store, assumes the same principles known from the world of physical stores, transferring the S-O-R model from physical to virtual outlets.
The influence of multiple store environment cues on perceived merchandise value and patronage intentions	Baker et al., 2002	This is one of the few studies to examine the simultaneous impact on consumers of multiple store environment cues, both in interaction with merchandising or point-of-sale artifacts and with store atmospherics and other interpersonal factors, all combined.
Effect of store design on consumer purchases: an empirical study of on-line bookstores	Liang and Lai, 2002	This study explores the importance of good design in an e-store, as in a physical store, and contends that after-sales support is equally important in both on- and offline environments.
Nuevas tendencias en merchandising: Generar experiencias para conquistar emociones y fidelizar clientes (New trends in merchandising: Generating experiences to conquer emotions and retain customers)	Zorrilla, 2002	A comprehensive model: relationship with the environment (exterior design, ambient conditions, interior design and social dimension), internal and behavioral responses.
Pre-purchase online information seeking: Search versus browse	Detlor et al., 2003	Real users in an exploratory study are asked to assess the importance of ease of browsing and searching for certain information on products in online stores designed for this purpose.
Consumer adoption of the Internet: The case of apparel shopping	Yoh et al., 2003	Theory of reasoned action: belief-attitude correlations to explain intention to purchase apparel online. Prior experience with the internet and shopping for apparel predicts intention to buy.
E-Scapes: The electronic physical environment and service tangibility	Koernig, 2003	Defines e-scape concept. Increased tangibility of atmospheric cues elicits more positive evaluations and reduces perceived risk.
Navigational web atmospherics. Explaining the influence of restrictive navigation cues	Dailey, 2004	The S-O-R paradigm is used to suggest that atmospheric cues influence consumers by altering their cognitive and affective states, which in turn influence their behavior (approach/avoidance) toward an e-store. Such behaviors include browsing/not browsing the site and revisiting it or otherwise.
The impact of consumer internet experience on channel preference and usage intentions across the different stages of the buying process	Frambach et al., 2007	This study investigates the different stages of the buying process in the case of a complex service with a long gestation period, where the pre-purchase, purchase and post-purchase stages are well defined and conditioned by both internet experience and channel preference.
Análisis del consumo virtual bajo la influencia de las dimensiones constituyentes de la experiencia web (Analysis of virtual consumption under the influence of the constituent dimensions of websperience)	Lorenzo et al., 2008	This study empirically analyses the main dimensions which, as shown in the literature, form the customer’s online experience or “websperience” (i.e., usability, interactivity, trust, aesthetic aspects and marketing mix)
Reciprocal effects between multichannel retailers’ offline and online brand images	Kwon and Lennon, 2009	A significant early study of the omnichannel concept, analyzing possible interferences and reciprocal effects in brand image across on- and offline channels.
Customer experience management in retailing: Understanding the buying process	Puccinelli et al., 2009	A review of the existing literature on consumer behavior and the importance of studying it across the various stages of the buying process so as to maximize satisfaction and relevance.
Online visual merchandising (VMD) cues and consumer pleasure and arousal: Purchasing versus browsing situation	Ha and Lennon, 2010	Though this experiment cannot be extrapolated to both genders, as it used only feedback from a female student population, it is notable in being one of the few studies to examine the influence of online merchandising cues on experience and intention to purchase in web store design (in this case for apparel)
E-atmosphere, emotional, cognitive, and behavioral responses	Kim and Lennon, 2010a, b	On the same population as the above study, and again in the apparel sector, this study shows the positive effect of a type of web merchandising, specifically a fitting room with a virtual human model, on experience and intention to buy.
Consumer responses to online atmosphere: The moderating role of atmospheric responsiveness	Ha and Lennon, 2011	The authors point to the significance of visual cues in online store design, as unlike with physical stores, the former are confined almost exclusively to this type of sensory cue, and are more dependent on consumers’ receptiveness to them.
Impact of virtual brand experience on purchase intentions: The role of multichannel congruence	Gabisch and Gwebu, 2011	This study explores the concept of multichannel congruence, discussing the implications of consistency across online and offline experiences of the same brand and how the brand’s image may be altered by different perceptions in each case.
An integrative review of sensory marketing: Engaging the senses to affect perception, judgment and behavior	Krishna, 2012	This study focuses on sensory marketing, also known as neuromarketing. The paper reviews the five senses and the importance for marketing of their being aroused.
Does more mean better? An examination of visual product presentation in e-retailing	Song and Kim, 2012	This study notably adapts the S-O-R model (initially conceived for a physical store environment) to e-stores, showing the importance of visual product presentation on consumers’ ultimate perception of the product and their patronage intention.
Shopper marketing 2.0: Opportunities and challenges. Shopper Marketing and the role of in-store marketing	Shankar, 2014	A review of recent trends in research on in-store marketing and the effects of new tech on the various stages of the path to purchase.
El papel de la vivacidad de la información online, la necesidad de tocar y la auto-confianza en la búsqueda de información online-offline (The role of vividness in online information, the need to touch and self-confidence in searching for information on- or offline)	Gurrea and Sanclemente, 2014	This study gauges the impact of vividness of information both in online interaction with the product and in the need to touch, and both this and vividness of information determine changes in experience when browsing a web environment.
The impact of the multi-channel retail mix on online store choice: Does online experience matter?	Melis et al., 2015	This research explores the importance of prior shopping experience in physical stores when shopping in e-stores and how consumer experience in one channel crosses over to the other.
An exploratory study on visual merchandising of an apparel store utilizing 3D technology	Baek et al., 2015	This study considers how physical stores are adopting visual techniques more associated with virtual environments such as merchandising items using 3D imagery.
Retailing in social virtual worlds: Developing a typology of virtual store atmospherics	Hassouneh and Brengman, 2015	This study anticipates future lines of research on online social interaction with the presence of other e-consumers interrelating within a web store as would occur in any real-world store.
A social commerce investigation of the role of trust in a social networking site on purchase intentions	Hajli et al., 2017	This study is based on the correlation between greater consumer trust in a website and purchase intention on that site. The research discusses the importance of other shoppers’ testimonials or experience on the site and of social networking to spread that trust.
Managing the visual environment of a fashion store: effects of visual complexity and order on sensation-seeking consumers	Jang et al., 2018	This interesting experiment recreates various degrees of complexity in the atmospherics of a virtual store and considers participants’ approach or avoidance response with variables relating to “environmental order” and the “amount of varied merchandise.”
Warm Hearts and Cool Heads: Uncomfortable Temperature Influences Reliance on Affect in Decision-Making	Hadi and Block, 2019	Study in the Journal of the Association for Consumer Research showing that “store temperature affects consumer decisions and behavior.”

## Author Contributions

Both authors listed have made a substantial, direct and intellectual contribution to the work, and approved it for publication.

## Conflict of Interest

The authors declare that the research was conducted in the absence of any commercial or financial relationships that could be construed as a potential conflict of interest.

## Footnotes

1 In 1998 Pine and Gilmore defined “immersion” as being part of the experience (e.g. playing a videogame in the first person).

2 Last year a study appeared in the Journal of the Association for Consumer Research showing that “store temperatures affect consumer decisions and their behavior When in a colder space (especially if this means one feels somehow uncomfortable), decisions are taken with more affective than rational processing. In other words, less thought and much less calculation is given to what is to be bought and why, and consumers are carried away by impulse.”
